# The Possible Role of Non-Structural Carbohydrates in the Regulation of Tree Hydraulics

**DOI:** 10.3390/ijms21010144

**Published:** 2019-12-24

**Authors:** Martina Tomasella, Elisa Petrussa, Francesco Petruzzellis, Andrea Nardini, Valentino Casolo

**Affiliations:** 1 Department of Life Sciences, University of Trieste, 34127 Trieste, Italy; fpetruzzellis@units.it (F.P.); nardini@units.it (A.N.); 2 Department of Agriculture, Food, Environmental and Animal Sciences, University of Udine, Via delle Scienze 91, 33100 Udine, Italy; elisa.petrussa@uniud.it (E.P.); valentino.casolo@uniud.it (V.C.)

**Keywords:** NSC, PLC, xylem sap, pH, hydraulic recovery, drought, stem, sugars, starch, embolism

## Abstract

The xylem is a complex system that includes a network of dead conduits ensuring long-distance water transport in plants. Under ongoing climate changes, xylem embolism is a major and recurrent cause of drought-induced tree mortality. Non-structural carbohydrates (NSC) play key roles in plant responses to drought and frost stress, and several studies putatively suggest their involvement in the regulation of xylem water transport. However, a clear picture on the roles of NSCs in plant hydraulics has not been drawn to date. We summarize the current knowledge on the involvement of NSCs during embolism formation and subsequent hydraulic recovery. Under drought, sugars are generally accumulated in xylem parenchyma and in xylem sap. At drought-relief, xylem functionality is putatively restored in an osmotically driven process involving wood parenchyma, xylem sap and phloem compartments. By analyzing the published data on stem hydraulics and NSC contents under drought/frost stress and subsequent stress relief, we found that embolism build-up positively correlated to stem NSC depletion, and that the magnitude of post-stress hydraulic recovery positively correlated to consumption of soluble sugars. These findings suggest a close relationship between hydraulics and carbohydrate dynamics. We call for more experiments on hydraulic and NSC dynamics in controlled and field conditions.

## 1. Introduction

The maintenance of long-distance water transport is an essential requisite for the survival and competitive success of vascular land plants. This is because plants lose large amounts of water through open stomata, parallel to CO_2_ uptake for photosynthesis. At the same time, plants need to keep tissues hydrated in order to grow and maintain metabolic processes [1].

Under ongoing climate changes apparently leading to increased intensity and frequency of drought events [2], hydraulic failure, i.e., the loss of substantial hydraulic function that induces plant desiccation, is a major and recurrent cause of tree mortality and forest decline worldwide [3]. This is explained by the fact that woody plants are vulnerable to embolism formation and apparently operate close to thresholds for runaway embolism in all biomes, even under ‘normal’ climate conditions [4]. Even forest biomes not assumed to be water-limited, such as tropical rainforests, have been reported to be more vulnerable to climate change than previously expected and related episodes of tree mortality were attributed to hydraulic deterioration [5].

Stems, branches and coarse roots are responsible for the transport of water from fine roots to the leaves and are also the main non-structural carbohydrate (NSC) storage sites in woody plants [6]. In woody tissues, the most abundant NSC specimens, mainly stored in parenchyma cells, are starch (the main insoluble storage compound) and low molecular weight sugars (glucose, fructose and sucrose). In some tree families, oligosaccharides such as stachyose and raffinose (sugars involved in phloem transport together with sucrose), and polyols such as pinitol, are also relevant and abundant [7,8]. Lipid stores are also particularly abundant in some *genera* such as *Tilia, Pinus, Picea* and *Larix* [9]. NSC stores play several key roles in plant functioning, but drought may alter the total amount, the allocation and the partitioning of different NSCs [6]. NSC depletion occurs when carbon consumption overcomes carbon assimilation, owing to drought-induced stomatal closure and/or increased respiration rates. Although not ubiquitous like hydraulic failure, carbon starvation has been observed in declining trees [10], and it does frequently occur in combination with hydraulic failure [3]. This underlines that the two mechanisms are not mutually exclusive and suggests possible close relationships between the two phenomena [11,12,13].

Together with drought resistance, the post-drought functional recovery potential of plant species may be a key driver of ecosystem dynamics under the predicted increase in frequency of drought events [1]. Nevertheless, while functional and physiological mechanisms adopted by plants during drought stress have been quite extensively explored, relatively less attention has been paid to tree recovery [14]. Trees surviving severe drought need to rebuild new organs and tissues, and new functional xylem [15]. Nevertheless, the possible legacy effects of severe droughts on long-lived woody plants include slowing down of growth for one to several years after the drought event, thus lowering the carbon storage capacity of forest ecosystems [16]. In particular, climate change may negatively impact plant growth because enhanced carbon fixation due to increased atmospheric [CO_2_] can possibly be counteracted by a higher increase in respiration rates due to rising temperatures [17]. Moreover, hydraulic acclimation can lead to reduction in growth and production of smaller, less conductive xylem conduits [18]. In addition, there is growing evidence that at least some tree species can recover their hydraulic function in the short term (hours to several days), when xylem water potential rises again close to zero [19]. Either if occurring through cambial growth or via restored conduit functionality, hydraulic recovery putatively implies the use of NSC stores [20]. Therefore, maintaining relatively high NSC levels during drought stress and keeping them available during the recovery phase may be crucial for tree vigor and survival.

In the past couple of decades, an increasing number of studies have focused on the role of NSCs in promoting plant resistance and resilience to drought stress. In fact, NSC-containing parenchyma cells and water-transporting xylem conduits are physically connected and can exchange water and solutes. However, an exhaustive portrait of the mechanistic relevance of NSCs for the hydraulic functioning of woody plants has not been provided yet [21], especially concerning organs with high construction costs such as stems and branches. In addition, a collection of the currently available studies relating stem embolism dynamics with the relative NSC changes is missing. The main goals of this review are (i) to provide a view on the state-of-the-art on the role of NSCs in the maintenance of water transport in woody plants under drought and recovery; and (ii) to identify possible relationships between tree hydraulic and NSC dynamics.

## 2. Plant Hydraulics and Drought Stress

Water absorbed by plant roots is pulled through the xylem up to the evaporative surfaces of leaf mesophyll cell walls in a passive process that does not require metabolic energy [22]. This process is explained by the Cohesion–Tension Theory, which in short indicates that plants generate a transpiration-driven “tension” to pull water and dissolved solutes upwards. The cohesive properties of the hydrogen-bonded water molecules, together with small lumen size and adhesive properties of conduits, allow to sustain large water tensions and, consequently, to avoid the breakage of the water continuum along the xylem pipeline [23,24]. This long-distance water transport occurs through a network of non-living conduits with reinforced cell walls that can mechanically sustain large negative pressures [25]. Xylem conduits are interconnected by pits, i.e., microscopic regions where the conducting elements are lacking secondary wall. Therefore, only a middle lamella and two thin primary walls separate adjacent conduits at the pit level. These separations, called pit membranes, are composed by a dense matrix of cellulose microfibrils and other wall components that ensure water and solutes flow but at the same time protect conduits against gas entry [26]. When a conduit becomes gas-filled, pit membranes act as “safety valves” blocking the aspiration of gas bubbles into the adjacent functional water-filled conduits [27]. Given that gas-filled conduits are subjected to positive pressure while the water-filled ones are under tension, a gas phase can be aspirated through the pores of the pit membranes at critical tension values, causing embolism propagation. This limit mainly depends on pit membrane properties such as thickness and porosity in angiosperms, and torus-pit aperture overlap in conifers [26]. Moreover, the expansion of a gas bubble with a certain radius in a xylem conduit at a certain Ψ_x_ (xylem water potential) depends on xylem sap surface tension (γ), which in turn depends on its chemistry and temperature [28]. At progressively lower Ψ_x_, more conduits will embolize, causing a reduction in xylem hydraulic conductance (usually quantified as percentage loss of hydraulic conductance, PLC). Embolism can be also caused by freeze-thaw events in winter. Because gases are insoluble in ice, when xylem sap freezes gas bubbles can coalesce and then expand when temperatures rise again and ice thaws, eventually causing the complete blockage of the conduit at rising xylem sap tension [29].

In response to drought stress, several tree species can ontogenetically modify their wood anatomical traits to reduce hydraulic vulnerability. These adjustments include decreased conduit dimensions (diameter and length), increased conduit wall thickness and wood density, and modifications of pit characteristics [25,30,31]. Even long-term hydraulic adjustments to previous year’s droughts can occur, as shown in potted saplings [18] and by dendrochronological studies in tropical rainforest trees [32]. On the other hand, prolonged and/or multiple droughts can weaken the resistance to xylem embolism through deterioration of inter-vessel pit membranes, a phenomenon known as “cavitation fatigue” [33]. Even though embolism formation is a purely physical process depending on the xylem structural properties described above, xylem functionality under changing water availability seems to be additionally connected to metabolic processes [34], involving also NSC metabolism.

## 3. Stem NSCs Dynamics and the Maintenance of Hydraulic Function under Drought

Wood is composed not only of xylem conduits (tracheids and vessels), but also of other cell types like ray and axial parenchyma and, for angiosperms, fibres. Parenchyma rays are the connection bridges between phloem and xylem, and allow the exchange of water and solutes between these two long-distance transport systems [35]. Ray parenchyma cells are connected to axial parenchyma cells, forming a 3D network that spreads to the xylem conduits [36]. Parenchyma cells are the primary sites of NSC storage in the sapwood [37]. In accordance with this, a strong positive correlation between wood parenchyma fraction and NSC content was found in young roots and stems of temperate tree species [38]. Fibres provide mechanical support to angiosperm woody stems, but in some species such as *Acer* and *Vitis,* the wider ones can also possess living protoplasts that can store and mobilize carbohydrates [39,40]. Among wood parenchyma cells, some of them are highly specialized and commonly referred to as vessel-associated cells (VACs [41,42]; sometimes called ‘contact cells’ [39]). VACs are in direct contact through pits with the dead conductive xylem conduits and fulfil several functions: water storage (sources of hydraulic capacitance [43]), water transport regulation through aquaporins [42,44], defence (e.g., through production of tyloses and gums [45]), osmoregulation and direct exchange of water, ions and other molecules such as soluble NSCs with the xylem sap ([42,46]; see Morris et al. [47] for a review). As an example, the export of sugars into the xylem apoplast may be important in winter to avoid or reduce the number of freeze-thaw embolism cycles, because sugars decrease the osmotic potential of xylem sap, thus lowering its freezing point [39,48]. In late winter, branch- and trunk-soluble sugars of *Pinus koraiensis* positively correlated with PLC and were overall more abundant than in summer [49]. This indicates that sugars mobilization over winter might represent a cold hardening strategy [48], but could be also putatively related to the need of maintaining and/or restoring hydraulic function [50,51] (see below).

NSCs are unequivocally involved in promoting plant drought resistance. Sugars, together with sugar alcohols, amino acids and inorganic ions, are accumulated for osmoregulation in living cells, including wood parenchyma and secondary phloem, in order to maintain cell turgor under declining water availability [52]. In fact, tropical tree seedlings enriched in NSCs maintained higher stem water potentials during severe drought and survived longer [53]. Moreover, given the physical continuity between phloem and xylem, insufficient soluble NSC contents in the secondary phloem could result in phloem turgor collapse and subsequent water leakage to the xylem [12,13,54].

Several woody species are known to perform stem photosynthesis in chloroplasts contained in bark, wood and pith. These species could be likely advantaged in the perspective of prolonged or recurrent drought, because stem photosynthesis re-assimilates CO_2_ released by mitochondrial respiration and by gaseous xylem efflux, thus becoming a local source of carbon under drought stress causing stomatal closure [55,56] and/or absence of leaves [57]. Sugars produced by woody tissue photosynthesis are suggested to play a role in the maintenance of tree hydraulic function under fluctuating water availability [58,59]. Moreover, in *Populus nigra* saplings, stem hydraulic vulnerability increased after prolonged shading of stems, likely reducing the stem NSC pool [60]. Albeit a convincing explanation for this phenomenon is not yet available, the authors suggested that NSCs produced by bark photosynthesis could be the substrate for the production and delivery of lipid surfactants into the xylem sap. Indeed, these molecules have been detected in xylem sap and are proposed to play a role in stabilizing gas nano-bubbles, therefore reducing the risks of embolism formation and propagation [61,62].

**Figure 1:**
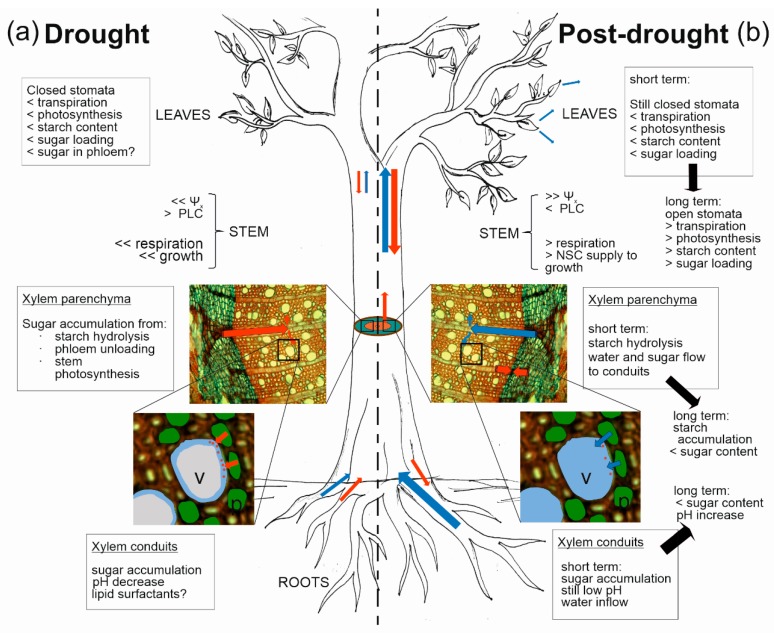
Schematic representation of putative roles of stem NSCs in the hydraulic regulation in woody plants. (**a**) During drought, root-to-leaf water transport is reduced and NSC reserves (manly from roots ad stems) are mobilized. Stomata are closed and photosynthesis is reduced, and hence sugar export from leaves and transport through phloem is limited. Decreased xylem water potential (Ψ_x_) induces embolism formation in xylem conduits and translocation of sugars to embolized vessels, putatively involving NSC metabolism in stem parenchyma cells and phloem unloading. (**b**) In the post-drought phase, restored water availability in the soil induces an increase in Ψ_x_ and a cascade of events leading to the refilling of previously embolized vessels. In the short term upon rehydration, stomata are still closed, transpiration is limited, sugars would still be loaded to the refilling vessels, and water would start to move in the direction of the refilling vessels, washing away the sugars accumulated in xylem sap. In the long term, stomata open and “normal” NSC metabolism and water relations are reestablished, and NSCs are invested for reactivation of cambial growth. Blue and red arrows indicate the direction of water and sugar fluxes, respectively, and their size indicates their magnitude. Question marks indicate putative processes. *v* = xylem vessel; *p* = wood parenchyma cells. < indicates low and > indicates high.

Multiple studies conducted in the last decade in different poplar species have demonstrated that severe drought triggers a cascade of metabolic processes in xylem parenchyma cells [63,64]. These are supposed to be early events involved in hydraulic restoration when water is again available to the plant [42,64] (see the following section). These events, for which we provide the sequence and related experimental proof below, apparently culminate in the accumulation of low molecular weight sugars in the xylem apoplast (Figure 1a).

Embolism formation is suggested to be sensed by the plant [65] and to act as a trigger for the activation of molecular signals connected to carbohydrate metabolism [42,65]. During the progression of drought and embolism formation, starch content in wood parenchyma cells was often reported to decrease [18,19,66,67]. This was associated with a contemporary increase of stem sucrose content [19,65,67] and with regulation of enzymes involved in starch synthesis and degradation, respectively [67]. Accordingly, the same pattern for starch and sucrose content was observed during winter embolism formation in *Juglans regia* [44] and embolism accumulation strongly correlated with starch depletion and with soluble sugars content in a conifer [49] and in an angiosperm [18].

Sucrose in wood parenchyma could originate from local starch hydrolysis as well as from phloem export. In xylem parenchyma of *Populus tremula x alba*, glucose, fructose and maltose derived from starch hydrolysis, while sucrose derived from long distance transport through the phloem-to-parenchyma rays pathway. All these sugars accumulated in the xylem apoplast and sap during drought, possibly due to the activation of plasma membrane sugar transporters in VACs [64]. In fact, sugar (especially maltose and sucrose) symporter genes were overexpressed parallel to starch degradation [64], likely sustaining the efflux of disaccharides to the cell walls in contact with xylem conduits. This was accompanied by a decrease in xylem sap pH [64,68], suggesting the involvement of sucrose-proton co-transporters. Indeed, acidification of xylem sap has been documented in several tree species during dry or prolonged frost periods [69,70]. A more acidic sap environment was then associated with an increase in acidic invertases activity, that would break the exported sucrose into glucose and fructose, which have been found in high concentrations in xylem sap of drought stressed plants [71]. Prior to this study, gene expression analyses showed up-regulation of ion transport, aquaporins and sugar metabolism in *Populus trichocarpa* [68,72], and similar patterns in carbon metabolism and aquaporin expression were found in grapevine petioles [73]. Some PIP1 and PIP2 aquaporin genes were overexpressed in parenchyma cells during drought [74,75], also in combination with changes in expression of genes related to sugar metabolism and transport [19]. Abscisic acid (ABA), which plays an important role in signaling drought and coordinating drought responses, is known to be involved in the regulation of stomatal conductance during physiological drought. Additionally, ABA is putatively suggested to affect carbohydrate partitioning by increasing the activity of β-amylases and invertases during drought not only in leaves [76], but also in stem wood, where a strong positive correlation between ABA content and hexoses has been found for *Populus nigra* [77].

## 4. Stem NSCs and the Post-Drought Recovery of Xylem Function

The recovery of hydraulic function following sub-lethal drought might represent a critical adaptive trait for long-lived trees facing recurrent and/or extreme drought spells [14]. A mid-to-long term strategy for hydraulic recovery is represented by the growth of new xylem by cambial activity, which requires the use of local or imported NSCs [15,18,78]. Moreover, during xylem development, an increasing demand of sugars (glucose, fructose and phosphate-sugars) for sustaining secondary wall formation [79] is fulfilled by phloem unloading into parenchyma rays and subsequent sugar movement via apoplastic and symplastic pathways [80].

Short-term mechanisms of xylem hydraulic restoration may act as a “first aid” strategy adopted by plants to partially restore water transport, enabling rehydration of shoots and increasing stomatal conductance. Those mechanisms include positive root pressure, stem pressure, water uptake through leaves and/or bark, and recovery under low residual xylem tension (namely refilling).

Positive root pressures typically generate at night or in early spring, when transpiration is almost entirely suppressed, and require fully saturated soil [81]. This phenomenon is commonly observed in herbaceous plants and arboreal monocots [82] but can also occur in adult temperate woody angiosperms before spring flush [78,83,84,85,86]. However, a dual mechanism including both generation of root pressure and stem osmotic pressure gradients was demonstrated in *Betula pendula* [87]. Sauter et al. [39] already observed starch depletion and an increase in sucrose concentration in the xylem sap, together with increased acid phosphatases and respiratory enzymes activity in “contact cells” (i.e., VACs) of *Acer saccharum* before spring flush. The authors hypothesised a metabolically active process involved in the restoration of sap flow in late winter [39]. Accordingly, in *J. regia* the positive xylem pressure associated with refilling in late winter/early spring was associated with high sugar concentrations in the xylem sap, indicating an osmotically driven process [88].

Evidence for vessel refilling under moderately negative xylem pressure has been provided in several plant species [8,19,40,67,85]. According to the existing model for refilling, VACs would be involved in the restoration of conduit functionality by generating an osmotic driving force and supplying water for refilling [20,42]. In this process, once water is available at recovery, the osmotic gradient generated by the accumulation of solutes in the xylem apoplast during drought reclaims water preferentially from VACs into the gas-filled vessels in an aquaporin-mediated process [20]. This fits with the in-vivo visualization of water droplets appearing and expanding in air-filled vessels in proximity of xylem parenchyma cells in grapevine [40,89,90]. Afterwards, once the vessels are refilled, ions and sugars in the xylem sap should be removed through the restored water flow [71].

This mechanistic model involves soluble NSCs in xylem sap as osmoticum for hydraulic recovery, but their possible additional roles are still not well understood. Sugar compounds located in xylem sap are transported even at long distances from the point of secretion into the apoplast and, especially when photosynthesis is reduced (e.g., in early spring before bud break), they can be accumulated and allocated via the transpiration stream to the leaves [91]. This could be an important carbon source during drought, but also upon rehydration when the leaf photosynthetic carbon fixation is low because stomata are still closed and/or photosynthesis is temporarily inhibited. In fact, the daily amount of sugars transported through the xylem from roots to shoots can exceed the daily amount of photosynthetically fixed CO_2_ under certain environmental conditions [91].

Hydraulic recovery is an energy-requiring process. Plasma membrane H^+^-ATPase, which in spring were shown to have a high activity in VACs with respect to more distant parenchyma cells [92], could energize the sugar-proton co-transporters during drought and recovery. This could be supported by increased stem respiration, that has been observed in trees after re-irrigation [93]. Moreover, fusicoccin (an activator of H^+^-ATPase) and orthovanadate (an inhibitor of plasma K^+^- and H^+^-ATPase) applied in the stem enhanced and inhibited, respectively, xylem hydraulic recovery [41].

Together with the above-mentioned sugar transporters, H^+^-ATPase of VACs is involved in the acidification of xylem sap [92]. In *Picea abies* growing at the timberline, the spring recovery of embolism was associated to a drop in pH from 7.0 to 5.5 [94], and to an increase in xylem sap surface tension (γ) from 50 to 67 mN m^−1^, that in turn increased the resistance to embolism [70]. According to a physicochemical study, the increase in mono- and disaccharides concentration observed during stress in xylem sap (e.g., [64]), cannot alone be responsible for such a large increase in γ [95]. Moreover, a decrease in pH from 8 to 4.5 of a 10 mM KBr solution (similar [K^+^] of xylem sap), increases its γ by only about 2 mN m^−1^ (from 72 to 74 mN m^−1^ [96]). A decrease in γ in xylem sap is supposed to be induced by the presence of surfactants [70,97]. However, investigations of xylem sap composition and chemistry in relation to variations in γ in field studies are scarce [98] and should deserve more attention, also in relation to changes in non-surface active (inorganic ions, sugars, alcohols) and surface-active (surfactants) compound concentration in xylem sap during drought and recovery.

Given its connectivity with xylem tissue, phloem has been also suggested to be involved in the refilling process [41,65,98,99]. In accordance, phloem inactivation via partial or full girdling prevented starch hydrolysis in parenchyma rays [100] and reduced or inhibited refilling [42,67,100,101,102,103]. Xylary chloroplasts, locally furnishing sugars, are also thought to play a role in the refilling process [58,59,101].

Refilling can occur within few minutes to hours [85], but it may take longer in dependence on the severity of drought [64]. Indeed, after being exposed to severe drought inducing high PLCs, several woody species recover water potentials within hours after re-irrigation, while the recovery of gas exchange [102] and eventual hydraulic restoration can require days [63,64,103]. Hydraulic recovery may occur to a less extent or not occur at all, in dependence on the species and magnitude of drought stress and hydraulic damage. For example, the magnitude of hydraulic recovery in *Salix matsudana* branches soaked for 6 hours was lower in samples dehydrated to Ψ_x_ of −2.2 MPa than in those reaching −1.5 MPa and −1.9 MPa [101]. In a similar way, stems of *Fagus sylvatica* saplings reaching PLCs close to thresholds for hydraulic failure (85%) did not recover hydraulic function in the short term [18].

The reliability of sample preparation methods for destructive hydraulic conductivity measurements has been the centre of a recent debate [104,105,106], casting doubt on the occurrence of embolism repair under tension [107]. In the past two decades, magnetic resonance imaging (MRI) and X-ray micro tomography (micro-CT) have been used to detect in vivo embolism formation and repair and, possibly, to solve those debates. Some in vivo studies showed the occurrence of refilling under negative pressure (in *Vitis* spp. [40,89,90,108,109]; in *Acer rubrum* [110]), while others did not [111,112,113]. The advantage of these methods over destructive ones is the putative absence of artefacts induced by cutting stem/branch samples. However, the use of X-ray micro-CT can cause cellular damage and alter membrane stability [114], thus possibly inhibiting the refilling process. Clearly, additional efforts are needed to converge on non-controversial protocols and methods for in vivo detection of embolism repair under tension.

Several studies, mainly conducted in gymnosperms, demonstrated that branches can absorb water through leaves and/or bark to refill embolised conduits [50,101,115,116,117]. In late winter and early spring, when soil water was still frozen, branch hydraulic recovery in *P. abies* was associated to water absorption from melting snow and to a peak in PIP aquaporin abundance in needle endodermis and phloem [50]. Similar to angiosperms recovering from drought, refilling though needle water uptake in *Picea glauca* was favoured by aquaporins located in VACs [115] and Liu et al. [101] have recently shown that sugars produced by corticular photosynthesis enhanced water absorption in the bark of soaked branches and promoted xylem refilling.

At present, only a few studies have measured the sugar concentration dynamics in xylem sap during hydraulic recovery. In *S. matsudana* branches, an increase in ion and soluble sugar content was observed upon stem soaking that preceded refilling via bark water uptake [118]. Similarly, in *Populus tremula* × *alba*, sugars that increased in concentration in xylem sap at the peak of drought, later decreased to unstressed levels upon recovery of hydraulic function [64]. All these studies support the idea that refilling could be driven by an osmotic gradient, probably generated by sugar accumulation in xylem sap.

## 5. NSC-PLC Relationships: A Survey from Currently Available Data

Relatively few studies, mainly limited to potted plants grown under controlled conditions or cut branches, have investigated the possible mechanistic roles of NSCs in the maintenance and recovery of xylem hydraulics. To highlight eventual recurrent patterns in PLC-NSC dynamics under embolism formation and recovery, we analysed the output of experiments reported in the literature that included quantitative data on stem NSC content and PLC at the end of the stress and after stress relief. Literature search terms included “PLC”, “embolism”, “non-structural carbohydrate”, “NSC”, “recovery”, “drought” and we selected only studies and plant species for which a significant increase in PLC during stress with respect to control plants or to pre-stress conditions was detected (Table 1). We obtained a list of 25 species, among which 23 were angiosperms and only two were conifers (a tree, *P. abies*, and a shrub, *Pinus mugo*). Out of 13 studies, 6 (including 9 species) were performed in controlled potted conditions, two (including 13 species) analysed cut branches which were rehydrated by soaking them in water, and 5 (5 species) were conducted in the field. The PLC increase was caused by drought or winter frost (causing freeze-thaw cycles and soil frost), or induced by sample dehydration in the laboratory. Moreover, the analysed studies and species substantially differed in the PLC reached at the end of the stress event (range from 20% to 85%), as well as in the duration of stress exposure (from few hours for dehydrated branches, to months in case of trees in the field) and recovery (from 1 h to several months). In 7 out of 32 treatment groups included in the analysis (some experiments included more treatment groups within a species [103,119]), hydraulic recovery was not observed (see Table 1). Also regarding NSCs, the available data were heterogeneous: in some studies only starch was measured, in others the NSCs derived from the bulk stem tissue were measured, while in others bark and wood were separated, and in only three studies (and species) NSC analyses were performed in both bark and wood. Moreover, only two experiments included measurements of xylem sap NSC content, which could not therefore be considered in the analysis.

Statistical analyses were performed with R software (R Core Team, 2017). Correlations between NSC and PLC were tested using Pearson’s and Spearman’s correlation tests (α = 0.05) for normally and non-normally distributed variables, respectively. For some correlations, stem PLC and/or NSC content were not available and, therefore, were not included in the analyses (parameters and data for analyses are reported in the Supplementary Materials; average values of parameters for each study were used). The results of the analyses are presented in Appendix A as Table A1 (correlations during the drought phase) and Table A2 (correlations during the recovery phase).

**Figure 2:**
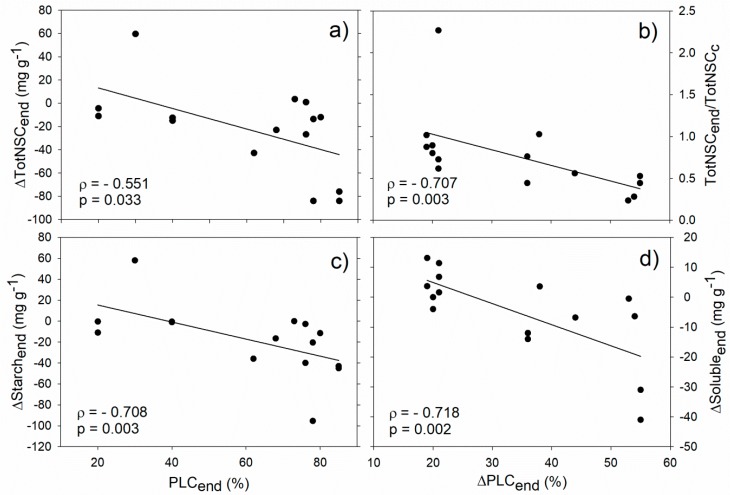
Relationships between stem hydraulics and non-structural carbohydrate (NSC) during drought/frost stress. Correlation between PLC at the end of stress (PLC_end_) and total NSC concentration (**a**, ΔTotNSC_end_) and starch (**c**, ΔStarch_end_) difference between drought/winter-stressed and non-stressed trees. Correlation between drought/frost-induced PLC increase (ΔPLC_end_) and the fraction of total NSC at the end of drought/winter stress with respect to control plants (**b**, TotNSC_end_/TotNSC_c_), and the soluble sugar difference between drought/winter-stressed and non-stressed trees (**d**, ΔSoluble_end_). Spearman’s correlation coefficient (ρ), related p-value and regression lines are reported.

Our analysis revealed significant correlations between stem hydraulics and NSCs during the drought/frost stress phase. In particular, PLC at the end of the stress (PLC_end_) negatively correlated with starch and total NSC concentration difference between drought/frost-stressed and non-stressed control trees (ΔStarch_end_ and ΔTotNSC_end_, respectively, Figure 2a,c). In almost all the analysed studies, NSCs were depleted during drought (i.e., ΔStarch_end_ and ΔTotNSC_end_ were negative) and this drop was particularly marked for plants with PLCs above 70–80%. Moreover, negative correlations were found between the PLC increase observed during drought (ΔPLC_end_) and both the fraction of total NSC at the end of drought/frost stress with respect to non-stressed trees (TotNSC_end_/TotNSC_c_), and the soluble sugar difference between drought/frost-stressed and non-stressed trees (ΔSoluble_end_, Figure 2b,d). All these correlations support the hypothesis that hydraulic and NSC dynamics under stress conditions are interdependent [49], and that hydraulic deterioration is generally, albeit not always, accompanied by NSC depletion [3,10].

**Figure 3:**
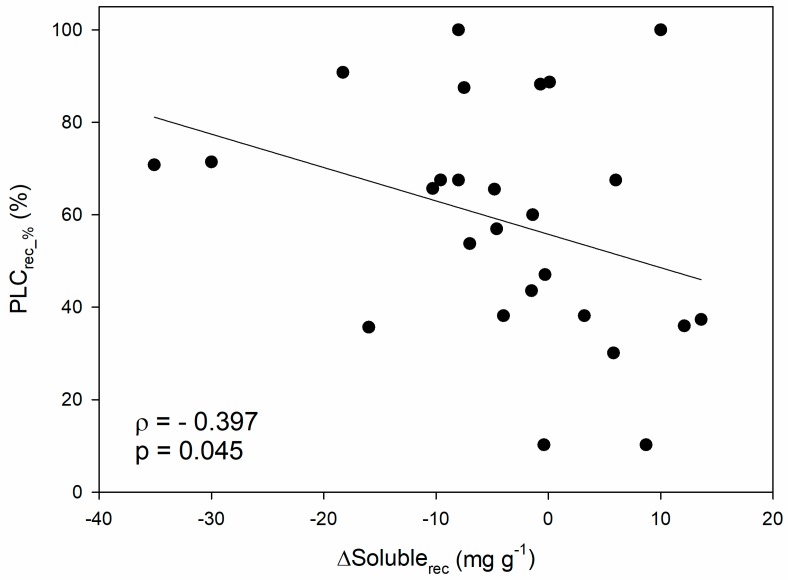
Relationship between percentage of recovered PLC (PLC_rec_%_) and the difference in soluble sugar content between recovery and end-drought/frost phase (ΔSoluble_rec_). Spearman’s correlation coefficient (ρ), related p-value and regression line are reported.

When looking at the hydraulic recovery phase, a negative correlation emerged between the recovered PLC (i.e., the PLC accumulated during stress that has been recovered upon stress relief, expressed in percentage, PLC_rec_%_) and the variation in soluble sugar content after recovery with respect to the end of the stress (ΔSoluble_rec_; *ρ* = −0.40; *p* < 0.05; Figure 3). In other words, hydraulic recovery positively correlates with the depletion of soluble NSCs during rehydration, and this confirms at a more general scale the pattern reported in a recent study conducted in 12 different angiosperm species [120], that was indeed included in the dataset. Moreover, this would be in agreement with the fact that the magnitude of hydraulic recovery in saplings of four angiosperm species depended on the available sugars at the end of drought [121].

These patterns suggest that the magnitude of hydraulic recovery could depend on the amount of soluble sugars available. As indicated in the previous chapter, the available sugars would play a pivotal role in the recovery because they would be demanded as a substrate for stem respiration [92], as osmotica for embolism repair and for building new xylem tissue. This finding would be also in agreement with the hypothesis that sugars are released into the vessels and subsequently removed when xylem sap flow is restored [42].

Until now, only a few studies have investigated within a species the importance of NSCs in the restoration of hydraulic function upon drought stress relief, by manipulating stem NSC content [103,119]. In one of these, in particular, it was shown that *Fraxinus ornus* plants with depleted stem wood NSCs at the end of a drought stress did not recover xylem hydraulics at re-irrigation, while those which maintained high NSC concentrations were able to do so [119]. This finding provides evidence in favour of the involvement of NSCs in tree hydraulic regulation and highlights that applying NSC manipulation in future studies could possibly provide evidence in favour or against the proposed hypotheses related to hydraulic recovery processes.

In our survey we could not identify any relationship between hydraulic parameters and total NSCs or starch contents in the recovery phase. The stored starch content pools in stems are strictly dependent on the species-specific stem anatomical features such as wood parenchyma fraction [120], phenology, period of the year, length of the stress and in general by the environmental stress conditions previously faced by the plant [118,125]. Moreover, in some species lipids are accumulated instead of starch [9,124]. Variability in starch dynamics in relation to hydraulic dynamics can be exemplified by a few examples collected from the studies included in the data survey. In apple trees, xylem embolism that accumulated in autumn-winter months was later repaired in spring, and this was accompanied by starch depletion in the stems [122]. However, in *P. abies* and *P. mugo* hydraulic recovery in late winter via needle/branch water uptake was observed in combination with an increase in starch content in the phloem, whereas in summer the recovery of embolism formed in May in *P. abies* was accompanied by a decrease in starch content [53,124]. In potted saplings of the same species subjected to drought in a greenhouse, hydraulic recovery was accompanied by starch increase in bark but not in wood [8]. It must be, however, underlined that the available data are extremely heterogeneous in terms of tissue analyzed, drivers of embolism formation (winter-induced, summer drought, bench dehydration), length of stress and, especially, recovery period (see Table 1). NSC responses observed at recovery are likely to depend on the time interval between the occurrence of the rain event after drought and the timing of sample collection. This is justified by the fact that short-term and long-term NSC dynamics are different, and may depend on the speed at which drought stress relief occurs, that in turn influences NSC-affecting processes such as stomata reopening, photosynthesis and phloem transport restoration (see Figure 1b).

## 6. Conclusions and Future Perspectives

Although several studies have underlined the importance of NSCs for maintaining hydraulic integrity under fluctuating water availability, a complete mechanistic picture of the processes involved is still lacking. In the light of the putative mechanisms of hydraulic regulation summarized in this review, we recognize the urgent need to design experiments in both controlled and field conditions that include the analysis of xylem sap, wood and bark NSC separately, and that in parallel quantify embolism during both drought and drought relief. In controlled greenhouse experiments, a possible approach that could shed light on role of NSCs in the plant hydraulic regulation is the manipulation of available NSCs in stems/roots, by coupling prolonged drought with, e.g., shading [53,60,119] and by looking at the effect of NSC variations in the different stem compartments (xylem sap, bark, wood) on plant hydraulics. An important issue is the general lack of field experiments on PLC and NSC patterns during drought and recovery periods. In the light of the current trends of climate change-driven forest decline observed in several ecosystems, there is an urgent need to understand the effects of repeated droughts accompanied by heat waves on tree hydraulic and NSC deterioration.

Considering that model species have been mainly used to study the mechanisms involved, it is still unclear to which extent results hold true for other species. Indeed, the current model of embolism refilling has been extensively tested only in some species of the genus *Populus* (see [42] for details), but it should be validated in a broader range of species. Routinely adding NSC measurements to hydraulic measurements, by using standardized protocols for NSC sampling and analysis [126], could shed light onto possible connections between the hydraulic and carbohydrate components. Moreover, given the possible diel variations in NSC contents in different organs and positions in the trees [127], the sampling time for NSC analysis is also crucial for comparisons between studies. A timely monitoring of sugars in sap, together with NSCs in wood and bark, coupled with hydraulic measurements, is needed in order to elucidate whether possible common patterns exist across species.

As underlined in the previous section, it is clear how technically difficult is to relate environment-driven NSC dynamics to the plant water status. Changes in solutes concentration during drought and recovery are likely to occur over small spatial and temporal scales [42]. Therefore, the gaps in knowledge on NSC dynamics in plants could be filled in the future by technological advances, including micro-scale localization of starch and sugar dynamics in parenchyma cells and non-functional xylem sap. A promising progress in this direction has been made by the use X-ray micro-CT in combination with a machine-learning algorithm for in vivo quantification of starch dynamics in stems of grapevine [128].

A further challenge would be to understand if sugars and other organic compounds in xylem sap could play a role in preserving the hydraulic integrity during fluctuations in water availability and, generally, under environmental stress conditions.

## Figures and Tables

**Table 1:** List of studies investigating embolism and non-structural carbohydrate dynamics under drought/frost and post-drought/frost recovery.

Species	Type of Sample	Embolism Induction	Rehydration Type	PLC before Recovery	NSC at Peak Embolism	NSC at Drought Relief	Recovery Duration	Hydraulic Recovery	Citation
*Fagus sylvatica*	Pot	SD	SR	85%	*St* decrease, *SS* increase in W and B	*St* increase, *SS* decrease in W and B	One week	No	[18]
*Fraxinus ornus*	Pot	SD	SR	76%	*SS* and *Tot* depleted only in B	Not changed	One day	Yes	[119]
				78%	*St* and *Tot* depleted in B and W	Not changed		No	
*Hibiscus glaber*	Field	SD	SR	70%	*SS* increase and *St* decrease	*SS* decrease and *St* increase	One week	Yes	[93]
*Ligustrum micranthum*				40%	*SS* increase and *St* decrease	Not changed	One week	Yes	
*Populus tremula x alba*	Pot	SD	SR	80%	*SS* increase in xylem sap *St* decrease, *Glu* increase in W	*SS* decrease in xylem sap *Glu* decrease in W	One week	Yes	[64]
*Laurus nobilis*	Pot	SD	SR	23%	Not changed	Not changed	One day	Yes	[103]
				34%	Not changed	Not changed	One day	Yes	
							One week	Yes	
*Arbutus unedo*	CB	BD	B/L WU	~50%	N.A.	*St* and *SS* decrease	One hour	Yes	[120]
*Ceratonia siliqua*						*St* increase, *SS* decrease		Yes	
*Cercis siliquastrum*						*St* and *SS* decrease		Yes	
*Eucalyptus camaldulensis*						*St* increase, *SS* decrease		No	
*Laurus nobilis*						*St* increase, *SS* decrease		Yes	
*Morus alba*						*St* increase, *SS* decrease		Yes	
*Myrtus communis*						*St* increase, *SS* decrease		Yes	
*Nerium oleander*						*St* decrease, *SS* increase		No	
*Olea europea*						*St* and *SS* decrease		Yes	
*Phillyrea latifolia*						*St* and *SS* increase		No	
*Pistacia lentiscus*						*St* and *SS* increase		No	
*Quercus ilex*						*St* and *SS* not changed		Yes	
*Salix matsudana*	CB	BD	B/L WU	NA	N.A.	*SS* increase, *St* decrease in B and W	6 hours	Yes	[101]
*Quercus pubescens*	Pot	SD	SR	73%	Increase in *SS* *St* not changed	N.A.	5 days	Yes	[121]
*Prunus mahleb*		SD	SR	30%	*SS* not changed Increase in *St*	N.A.		No	
*Robinia pseudoacacia*		SD	SR	68%	Decrease in *St* and *SS*	N.A.		No	
*Ailanthus altissima*		SD	SR	62%	Decrease in *St* and *SS*	N.A.		Yes	
*Malus domestica* var. Golden delicious	Field	FT + FD	SR	70%	*St* not changed in W and B	*St* decrease in W and B, more pronounced in W	1 to 3 months	Yes	[122]
*Malus domestica* (4 cultivars)	Field	FT + FD	SR	20–80%	N.A.	*St* increase in W and B	Several weeks	Yes	[123]
*Picea abies*	Field	FT + FD	B/L WU	43%	*SS* not changed Very low, constant *St* in W and B	*St* increase in phloem and needles *SS* not changed Very low *St* in W	1 month	Yes	[50]
		SD	SR	30%	*St* increase in phloem	*St* decrease in phloem	3 months	Yes	
*Picea abies*	Pot	SD	SR	20%	*St* depletion in B *Tot* not changed in B and W	30% depletion of *Tot* in W	One week	Yes	[8]
*Pinus mugo*	Field	FT + FD	B/L WU	40%	*St* and *SS* not changed	*St* increase only in phloem and needles *SS* not changed	One month	Yes	[124]

Pot = pot experiment; Field = field experiment; CB = cut branches; SD = soil drought; FT = freeze-thaw; FD = frost drought; BD = bench dehydration; B/L WU = bark/leaf water uptake; SR, soil rehydration; *St* = starch; *SS* = soluble sugars; *Glu* = glucose; *Tot* = total NSC; B = bark; W = wood; BT = bulk tissue; N.A. = not available data.

## Supplementary Materials

Supplementary materials can be found at https://www.mdpi.com/1422-0067/21/1/144/s1.

## Abbreviations

PLC	Percentage loss of hydraulic conductance
NSC	Non-Structural Carbohydrates

## Appendix A

**Table A1:** Pearson’s/Spearman’s correlation coefficients between hydraulic and non-structural carbohydrate parameters during drought/winter stress.

	Soluble_end_	Starch_end_	TotNSC_end_	ΔSoluble_end_	ΔStarch_end_	ΔTotNSC_end_	Soluble_end_/Soluble_c_	Starch_end_/Starch_c_	TotNSC_end_/TotNSCc
ΔPLC_end_	−0.273	−0.196	−0.324	−**0.718** **	−0.288	0.417	−**0.592** *	0.270	−**0.707** **
PLC_end_	0.167	0.216	0.088	0	−**0.708** **	−**0.551** *	−0.048	−0.142	−0.473 ^(^*^)^
PLC_rec_%_	/	/	/	−**0.555** *	0.409	0.254	−0.306	−0.348	0.086

Significance of correlations (*p* < 0.05) is indicated in bold. ^(^*^)^ 0.05 < *p* < 0.10; * 0.01 < *p* < 0.05; ** *p* < 0.01.

**Table A2:** Pearson’s/Spearman’s correlation coefficients between hydraulic and non-structural carbohydrate parameters at recovery.

	Soluble_end_	Starch_end_	TotNSC_end_	ΔSoluble_rec_	ΔStarch_rec_	ΔTotNSC_rec_	Soluble_rec_	Starch_rec_	Tot_rec_	Soluble_rec_/Soluble_end_	Starch_rec_/Starch_end_	TotNSC_rec_/TotNSC_end_
ΔPLC_rec_	0.027	0.067	0.160	−0.287	−0.172	0	−0.067	−0.009	0.297	−0.201	0.160	0.276
PLC_rec_%_	0.054	−0.284 ^(^*^)^	−0.062	−**0.397** *	−0.131	−0.065	−0.170	−0.324 ^(^*^)^	−0.026	−0.181	0.249	−0.034

Significance of correlations (*p* < 0.05) is indicated in bold. ^(^*^)^ 0.05 < *p* < 0.10; * 0.01 < *p* < 0.05.
